# Supplementary material for: Evaluation of the Effect of the ENSO Cycle on the Distribution Potential of the Genus Anastrepha of Horticultural Importance in the Neotropics and Panama
Source: Insects. 2023 Aug 18;14(8):714. doi: 10.3390/insects14080714 (PMC10455666; doi:10.3390/insects14080714)
Supplement: Supplementary file 1 [file insects-14-00714-s001.zip › insects-2533089-supplementary.pdf]

Table S1. ***Anastrepha* spp. Species Database**

Species, Longitude, Latitude	Source	
A.grandis, -78.17000000000,8.76580000000	Tesis	http://up-rid.up.ac.pa/id/eprint/348
A.grandis, -80.00000000000,9.00000000000	CABI	https://www.cabidigitallibrary.org (last access March 3, 2022 CABI-1942-2020-5649)
A.grandis,-64.00000000000,-34.00000000000	CABI	https://www.cabidigitallibrary.org (last access March 3, 2022 CABI-1942-2020-5649)
A.grandis,-54.38440000000,-26.71440000000	GBIF	https://doi.org/10.15468/dl.62pb98
A.grandis,-65.00000000000,-17.00000000000	CABI	https://www.cabidigitallibrary.org (last access March 3, 2022 CABI-1942-2020-5649)
A.grandis,-64.38139000000,-22.71527900000	GBIF	https://doi.org/10.15468/dl.62pb98
A.grandis,-55.00000000000,-10.00000000000	CABI	https://www.cabidigitallibrary.org (last access March 3, 2022 CABI-1942-2020-5649)
A.grandis,-42.00000000000,-12.00000000000	CABI	https://www.cabidigitallibrary.org (last access March 3, 2022 CABI-1942-2020-5649)
A.grandis,-40.75000000000,-20.00000000000	CABI	https://www.cabidigitallibrary.org (last access March 3, 2022 CABI-1942-2020-5649)
A.grandis,-49.63623000000,-15.58071000000	CABI	https://www.cabidigitallibrary.org (last access March 3, 2022 CABI-1942-2020-5649)
A.grandis,-56.00000000000,-13.00000000000	CABI	https://www.cabidigitallibrary.org (last access March 3, 2022 CABI-1942-2020-5649)
A.grandis,-55.00000000000,-20.50000000000	CABI	https://www.cabidigitallibrary.org (last access March 3, 2022 CABI-1942-2020-5649)
A.grandis,-51.33333000000,-24.50000000000	CABI	https://www.cabidigitallibrary.org (last access March 3, 2022 CABI-1942-2020-5649)
A.grandis,-42.50000000000,-22.25000000000	CABI	https://www.cabidigitallibrary.org (last access March 3, 2022 CABI-1942-2020-5649)
A.grandis,-53.50000000000,-30.00000000000	CABI	https://www.cabidigitallibrary.org (last access March 3, 2022 CABI-1942-2020-5649)
A.grandis,-50.00000000000,-27.00000000000	CABI	https://www.cabidigitallibrary.org (last access March 3, 2022 CABI-1942-2020-5649)
A.grandis,-49.00000000000,-22.00000000000	CABI	https://www.cabidigitallibrary.org (last access March 3, 2022 CABI-1942-2020-5649)
A.grandis,-47.87643300000,-15.73301300000	GBIF	https://doi.org/10.15468/dl.62pb98

A.grandis,-42.36260000000,-21.98030000000	GBIF	https://doi.org/10.15468/dl.62pb98	
A.grandis,-47.13540000000,-23.53080000000	GBIF	https://doi.org/10.15468/dl.62pb98	
A.grandis,-57.29610000000,-25.31060000000	GBIF	https://doi.org/10.15468/dl.62pb98	
A.grandis,-73.25000000000,4.00000000000	CABI	https://www.cabidigitallibrary.org (last access March 3, 2022 CABI-1942-2020-5649)	
A.grandis,-75.76000200000,4.38000000000	GBIF	https://doi.org/10.15468/dl.62pb98	
A.grandis,-75.63418000000,6.15792000000	GBIF	https://doi.org/10.15468/dl.62pb98	
A.grandis,-74.29730000000,4.57090000000	GBIF	https://doi.org/10.15468/dl.62pb98	
A.grandis,-76.56980000000,3.37230000000	GBIF	https://doi.org/10.15468/dl.62pb98	
A.grandis,-78.25000000000,-1.25000000000	CABI	https://www.cabidigitallibrary.org (last access March 3, 2022 CABI-1942-2020-5649)	
A.grandis,-77.84018700000,-1.61747100000	GBIF	https://doi.org/10.15468/dl.62pb98	
A.grandis,-75.25000000000,-10.00000000000	CABI	https://www.cabidigitallibrary.org (last access March 3, 2022 CABI-1942-2020-5649)	
A.grandis,-71.40280000000,-12.89500000000	GBIF	https://doi.org/10.15468/dl.62pb98	
A.grandis,-58.00000000000,-23.33333000000	CABI	https://www.cabidigitallibrary.org (last access March 3, 2022 CABI-1942-2020-5649)	
A.grandis,-66.00000000000,8.00000000000	CABI	https://www.cabidigitallibrary.org (last access March 3, 2022 CABI-1942-2020-5649)	
A.grandis,-52.40416700000,-26.87694400000	SPECIES	http://www.splink.org.br/	speciesLink network, 16-Jun-2022 12:51, specieslink.net/search
A.grandis,-53.16805600000,-26.89388900000	SPECIES	http://www.splink.org.br/	speciesLink network, 16-Jun-2022 12:51, specieslink.net/search
A.grandis,-44.31810000000,-23.00670100000	SPECIES	http://www.splink.org.br/	speciesLink network, 16-Jun-2022 12:51, specieslink.net/search
A.grandis,-55.34456100000,-27.31669300000	CABI	https://www.cabidigitallibrary.org (last access March 3, 2022 CABI-1942-2020-5649)	
A.grandis,-54.74168000000,-26.57173400000	SPECIES	http://www.splink.org.br/	speciesLink network, 16-Jun-2022 12:51, specieslink.net/search
A.grandis,-58.01699600000,-31.39341300000	CABI	https://www.cabidigitallibrary.org (last access March 3, 2022 CABI-1942-2020-5649)	

A.grandis,-63.80600300000,-22.51753100000	GBIF	https://doi.org/10.15468/dl.62pb98
A.serpentina,-89.38999900000,18.51000000000	GBIF	https://doi.org/10.15468/dl.t653n6
A.serpentina,-95.16999800000,18.50000000000	GBIF	https://doi.org/10.15468/dl.t653n6
A.serpentina-100.94999700000,25.29999900000	GBIF	https://doi.org/10.15468/dl.t653n6
A.serpentina,-97.01000200000,18.92000000000	GBIF	https://doi.org/10.15468/dl.t653n6
A.serpentina,-89.64410000000,21.10213900000	GBIF	https://doi.org/10.15468/dl.t653n6
A.serpentina,-98.95939500000,18.90587600000	GBIF	https://doi.org/10.15468/dl.t653n6
A.serpentina,-89.89500000000,18.56444400000	GBIF	https://doi.org/10.15468/dl.t653n6
A.serpentina,-90.06083300000,18.97527800000	GBIF	https://doi.org/10.15468/dl.t653n6
A.serpentina,-98.66000000000,20.98777800000	GBIF	https://doi.org/10.15468/dl.t653n6
A.serpentina,-92.18300000000,14.81700000000	GBIF	https://doi.org/10.15468/dl.t653n6
A.serpentina-102.00000000000,23.00000000000	CABI	https://www.cabidigitallibrary.org (last access March 3, 2022 CABI 1934-2021 -5665)
A.serpentina,-83.56671400000,8.67909600000	GBIF	https://doi.org/10.15468/dl.t653n6
A.serpentina,-85.35234500000,10.34911900000	GBIF	https://doi.org/10.15468/dl.t653n6
A.serpentina,-84.60812600000,9.77649400000	GBIF	https://doi.org/10.15468/dl.t653n6
A.serpentina,-83.45916300000,9.81835700000	GBIF	https://doi.org/10.15468/dl.t653n6
A.serpentina,-85.54106900000,10.98316600000	GBIF	https://doi.org/10.15468/dl.t653n6
A.serpentina,-84.48510100000,10.20136100000	CABI	https://www.cabidigitallibrary.org (last access March 3, 2022 CABI 1934-2021 -5665)
A.serpentina,-85.05995200000,9.86709500000	GBIF	https://doi.org/10.15468/dl.t653n6
A.*serpentina,*-83.03070900000,9.66143800000	GBIF	https://doi.org/10.15468/dl.t653n6

A.serpentina,-85.38412300000,9.88585400000	GBIF	https://doi.org/10.15468/dl.t653n6
A.serpentina,-85.63368800000,10.13416800000	GBIF	https://doi.org/10.15468/dl.t653n6
A.serpentina,-85.02993400000,10.71888100000	GBIF	https://doi.org/10.15468/dl.t653n6
A.serpentina,-84.78362300000,10.44039400000	GBIF	https://doi.org/10.15468/dl.t653n6
A.serpentina,-84.31692400000,9.84894300000	GBIF	https://doi.org/10.15468/dl.t653n6
A.serpentina,-85.35191300000,10.77678400000	GBIF	https://doi.org/10.15468/dl.t653n6
A.serpentina,-82.79293600000,8.89147300000	GBIF	https://doi.org/10.15468/dl.t653n6
A.serpentina,-83.26379900000,8.97909600000	GBIF	https://doi.org/10.15468/dl.t653n6
A.serpentina,-84.00000000000,10.00000000000	GBIF	https://doi.org/10.15468/dl.t653n6
A.serpentina,-63.82000000000,-18.13999900000	GBIF	https://doi.org/10.15468/dl.t653n6
A.serpentina,-63.44683000000,-17.67657000000	GBIF	https://doi.org/10.15468/dl.t653n6
A.serpentina,-64.37243000000,-17.24656700000	GBIF	https://doi.org/10.15468/dl.t653n6
A.serpentina,-43.61000100000,-19.34000000000	GBIF	https://doi.org/10.15468/dl.t653n6
A.serpentina,-55.49150000000,-8.78320000000	GBIF	https://doi.org/10.15468/dl.t653n6
A.serpentina,-44.31580000000,-23.00570000000	GBIF	https://doi.org/10.15468/dl.t653n6
A.serpentina,-52.40000000000,-27.05000000000	GBIF	https://doi.org/10.15468/dl.t653n6
A.serpentina,-56.13500000000,-2.03770000000	GBIF	https://doi.org/10.15468/dl.t653n6
A.serpentina,-54.70090000000,-2.45060000000	GBIF	https://doi.org/10.15468/dl.t653n6
A.serpentina,-51.92530000000,-14.23500000000	GBIF	https://doi.org/10.15468/dl.t653n6
A.serpentina,-42.00000000000,-11.00000000000	GBIF	https://doi.org/10.15468/dl.t653n6

A.serpentina,-55.00000000000,-10.00000000000	CABI	https://www.cabidigitallibrary.org (last access March 3, 2022 CABI 1934-2021 - 5665)
A.serpentina,-52.00000000000,1.00000000000	CABI	https://www.cabidigitallibrary.org (last access March 3, 2022 CABI 1934-2021 - 5665)
A.serpentina,-64.50000000000,-3.75000000000	CABI	https://www.cabidigitallibrary.org (last access March 3, 2022 CABI 1934-2021 - 5665)
A.serpentina,-42.00000000000,-12.00000000000	CABI	https://www.cabidigitallibrary.org (last access March 3, 2022 CABI 1934-2021 - 5665)
A.serpentina,-40.75000000000,-20.00000000000	CABI	https://www.cabidigitallibrary.org (last access March 3, 2022 CABI 1934-2021 - 5665)
A.serpentina,-49.63623000000,-15.58071000000	CABI	https://www.cabidigitallibrary.org (last access March 3, 2022 CABI 1934-2021 - 5665)
A.serpentina,-45.00000000000,-5.00000000000	CABI	https://www.cabidigitallibrary.org (last access March 3, 2022 CABI 1934-2021 - 5665)
A.serpentina,-55.00000000000,-20.50000000000	CABI	https://www.cabidigitallibrary.org (last access March 3, 2022 CABI 1934-2021 - 5665)
A.serpentina,-44.00000000000,-18.00000000000	CABI	https://www.cabidigitallibrary.org (last access March 3, 2022 CABI 1934-2021 - 5665)
A.serpentina,-53.00000000000,-4.00000000000	CABI	https://www.cabidigitallibrary.org (last access March 3, 2022 CABI 1934-2021 - 5665)
A.serpentina,-36.50000000000,-7.25000000000	CABI	https://www.cabidigitallibrary.org (last access March 3, 2022 CABI 1934-2021 - 5665)
A.serpentina,-51.33333000000,-24.50000000000	CABI	https://www.cabidigitallibrary.org (last access March 3, 2022 CABI 1934-2021 - 5665)
A.serpentina,-37.75000000000,-8.33333000000	CABI	https://www.cabidigitallibrary.org (last access March 3, 2022 CABI 1934-2021 - 5665)
A.serpentina,-42.33333000000,-7.25000000000	CABI	https://www.cabidigitallibrary.org (last access March 3, 2022 CABI 1934-2021 - 5665)
A.serpentina,-42.50000000000,-22.25000000000	CABI	https://www.cabidigitallibrary.org (last access March 3, 2022 CABI 1934-2021 - 5665)
A.serpentina,-36.50000000000,-5.75000000000	CABI	https://www.cabidigitallibrary.org (last access March 3, 2022 CABI 1934-2021 - 5665)
A.serpentina,-53.50000000000,-30.00000000000	CABI	https://www.cabidigitallibrary.org (last access March 3, 2022 CABI 1934-2021 - 5665)
A.serpentina,-63.00000000000,-11.00000000000	CABI	https://www.cabidigitallibrary.org (last access March 3, 2022 CABI 1934-2021 - 5665)
A.serpentina,-61.25000000000,2.25000000000	CABI	https://www.cabidigitallibrary.org (last access March 3, 2022 CABI 1934-2021 - 5665)
A.serpentina,-50.00000000000,-27.00000000000	CABI	https://www.cabidigitallibrary.org (last access March 3, 2022 CABI 1934-2021 - 5665)

A.serpentina,-49.00000000000,-22.00000000000	CABI	https://www.cabidigitallibrary.org (last access March 3, 2022 CABI 1934-2021 -5665)
A.serpentina,-48.00000000000,-10.50000000000	CABI	https://www.cabidigitallibrary.org (last access March 3, 2022 CABI 1934-2021 -5665)
A.serpentina,-79.38999900000,-0.08000000000	GBIF	https://doi.org/10.15468/dl.t653n6
A.serpentina,-78.25000000000,-1.25000000000	CABI	https://www.cabidigitallibrary.org (last access March 3, 2022 CABI 1934-2021 -5665)
A.serpentina,-80.11000100000,8.39000000000	GBIF	https://doi.org/10.15468/dl.t653n6
A.serpentina,-79.51670000000,8.98330000000	GBIF	https://doi.org/10.15468/dl.t653n6
A.serpentina,-80.00000000000,9.00000000000	CABI	https://www.cabidigitallibrary.org (last access March 3, 2022 CABI 1934-2021 -5665)
A.serpentina,-76.60530000000,3.11710000000	GBIF	https://doi.org/10.15468/dl.t653n6
A.serpentina,-73.25000000000,4.00000000000	CABI	https://www.cabidigitallibrary.org (last access March 3, 2022 CABI 1934-2021 -5665)
A.serpentina,-58.42020000000,6.86030000000	GBIF	https://doi.org/10.15468/dl.t653n6
A.serpentina,-59.39980000000,3.20700000000	GBIF	https://doi.org/10.15468/dl.t653n6
A.serpentina,-59.00000000000,5.00000000000	CABI	https://www.cabidigitallibrary.org (last access March 3, 2022 CABI 1934-2021 -5665)
A.serpentina,-53.00000000000,4.00000000000	CABI	https://www.cabidigitallibrary.org (last access March 3, 2022 CABI 1934-2021 -5665)
A.serpentina,-88.75000000000,17.25000000000	CABI	https://www.cabidigitallibrary.org (last access March 3, 2022 CABI 1934-2021 -5665)
A.serpentina,-88.91667000000,13.83333000000	CABI	https://www.cabidigitallibrary.org (last access March 3, 2022 CABI 1934-2021 -5665)
A.serpentina,-90.25000000000,15.50000000000	CABI	https://www.cabidigitallibrary.org (last access March 3, 2022 CABI 1934-2021 -5665)
A.serpentina,-86.50000000000,15.00000000000	CABI	https://www.cabidigitallibrary.org (last access March 3, 2022 CABI 1934-2021 -5665)
A.serpentina,-85.00000000000,13.00000000000	CABI	https://www.cabidigitallibrary.org (last access March 3, 2022 CABI 1934-2021 -5665)
A.serpentina,-58.00000000000,-23.33333000000	CABI	https://www.cabidigitallibrary.org (last access March 3, 2022 CABI 1934-2021 -5665)
A.serpentina,-75.25000000000,-10.00000000000	CABI	https://www.cabidigitallibrary.org (last access March 3, 2022 CABI 1934-2021 -5665)

A.serpentina,-56.00000000000,4.00000000000	CABI	https://www.cabidigitallibrary.org (last access March 3, 2022 CABI 1934-2021 - 5665)	
A.serpentina,-66.00000000000,8.00000000000	CABI	https://www.cabidigitallibrary.org (last access March 3, 2022 CABI 1934-2021 - 5665)	
A.serpentina,-79.68131800000,9.30351800000	GBIF	https://doi.org/10.15468/dl.t653n6	
A.serpentina,-80.41422700000,8.63464100000	GBIF	https://doi.org/10.15468/dl.t653n6	
A.serpentina,-80.53070600000,8.35957900000	GBIF	https://doi.org/10.15468/dl.t653n6	
A.serpentina,-82.61458200000,9.49234400000	GBIF	https://doi.org/10.15468/dl.t653n6	
A.serpentina,-39.10110000000,-12.66100000000	SPECIES	http://www.splink.org.br/	speciesLink network, 16-Jun-2022 12:21, specieslink.net/se
A.serpentina,-65.13792500000,-26.68785800000	GBIF	https://doi.org/10.15468/dl.t653n6	
A.serpentina,-63.80600300000,-22.51753100000	GBIF	https://doi.org/10.15468/dl.t653n6	
A. obliqua,-84.13,9.39	GBIF	https://doi.org/10.15468/dl.93h5p7	
A. obliqua,-85.35,10.35	GBIF	https://doi.org/10.15468/dl.93h5p7	
A. obliqua,-84.99,10.61	GBIF	https://doi.org/10.15468/dl.93h5p7	
A. obliqua,-85.06,9.87	GBIF	https://doi.org/10.15468/dl.93h5p7	
A. obliqua,-85.73,10.90	GBIF	https://doi.org/10.15468/dl.93h5p7	
A. obliqua,-85.35,10.78	GBIF	https://doi.org/10.15468/dl.93h5p7	
A. obliqua,-84.41,9.97	GBIF	https://doi.org/10.15468/dl.93h5p7	
A. obliqua,-84.00,10.00	CABI	https://www.cabidigitallibrary.org (Last access March 3, 2022 CABI 1935-2019 - 5659)	
A. obliqua,-47.81,-21.17	GBIF	https://doi.org/10.15468/dl.93h5p7	
A. obliqua,-49.53,-25.46	GBIF	https://doi.org/10.15468/dl.93h5p7	
A. obliqua,-49.06,-25.44	GBIF	https://doi.org/10.15468/dl.93h5p7	

A. obliqua,-38.45,-5.13	GBIF	https://doi.org/10.15468/dl.9 3h5p7
A. obliqua,-38.46,-12.97	GBIF	https://doi.org/10.15468/dl.9 3h5p7
A. obliqua,-55.00,-10.00	CABI	https://www.cabidigitallibrary.org (Last access March 3, 2022 CABI 1935-2019 - 5659)
A. obliqua,-70.00,-9.00	CABI	https://www.cabidigitallibrary.org (Last access March 3, 2022 CABI 1935-2019 - 5659)
A. obliqua,-36.42,-9.58	CABI	https://www.cabidigitallibrary.org (Last access March 3, 2022 CABI 1935-2019 - 5659)
A. obliqua,-52.00,1.00	CABI	https://www.cabidigitallibrary.org (Last access March 3, 2022 CABI 1935-2019 - 5659)
A. obliqua,-64.50,-3.75	CABI	https://www.cabidigitallibrary.org (Last access March 3, 2022 CABI 1935-2019 - 5659)
A. obliqua,-42.00,-12.00	CABI	https://www.cabidigitallibrary.org (Last access March 3, 2022 CABI 1935-2019 - 5659)
A. obliqua,-39.50,-5.00	CABI	https://www.cabidigitallibrary.org (Last access March 3, 2022 CABI 1935-2019 - 5659)
A. obliqua,-47.75,-15.75	CABI	https://www.cabidigitallibrary.org (Last access March 3, 2022 CABI 1935-2019 - 5659)
A. obliqua,-40.75,-20.00	CABI	https://www.cabidigitallibrary.org (Last access March 3, 2022 CABI 1935-2019 - 5659)
A. obliqua,-49.64,-15.58	CABI	https://www.cabidigitallibrary.org (Last access March 3, 2022 CABI 1935-2019 - 5659)
A. obliqua,-45.00,-5.00	CABI	https://www.cabidigitallibrary.org (Last access March 3, 2022 CABI 1935-2019 - 5659)
A. obliqua,-56.00,-13.00	CABI	https://www.cabidigitallibrary.org (Last access March 3, 2022 CABI 1935-2019 - 5659)
A. obliqua,-55.00,-20.50	CABI	https://www.cabidigitallibrary.org (Last access March 3, 2022 CABI 1935-2019 - 5659)
A. obliqua,-44.00,-18.00	CABI	https://www.cabidigitallibrary.org (Last access March 3, 2022 CABI 1935-2019 - 5659)
A. obliqua,-53.00,-4.00	CABI	https://www.cabidigitallibrary.org (Last access March 3, 2022 CABI 1935-2019 - 5659)
A. obliqua,-36.50,-7.25	CABI	https://www.cabidigitallibrary.org (Last access March 3, 2022 CABI 1935-2019 - 5659)
A. obliqua,-51.33,-24.50	CABI	https://www.cabidigitallibrary.org (Last access March 3, 2022 CABI 1935-2019 - 5659)
A. obliqua,-37.75,-8.33	CABI	https://www.cabidigitallibrary.org (Last access March 3, 2022 CABI 1935-2019 - 5659)

A. obliqua,-42.33,-7.25	CABI	https://www.cabidigitallibrary.org (Last access March 3, 2022 CABI 1935-2019 - 5659)
A. obliqua,-42.50,-22.25	CABI	https://www.cabidigitallibrary.org (Last access March 3, 2022 CABI 1935-2019 - 5659)
A. obliqua,-36.50,-5.75	CABI	https://www.cabidigitallibrary.org (Last access March 3, 2022 CABI 1935-2019 - 5659)
A. obliqua,-53.50,-30.00	CABI	https://www.cabidigitallibrary.org (Last access March 3, 2022 CABI 1935-2019 - 5659)
A. obliqua,-63.00,-11.00	CABI	https://www.cabidigitallibrary.org (Last access March 3, 2022 CABI 1935-2019 - 5659)
A. obliqua,-61.25,2.25	CABI	https://www.cabidigitallibrary.org (Last access March 3, 2022 CABI 1935-2019 - 5659)
A. obliqua,-50.00,-27.00	CABI	https://www.cabidigitallibrary.org (Last access March 3, 2022 CABI 1935-2019 - 5659)
A. obliqua,-49.00,-22.00	CABI	https://www.cabidigitallibrary.org (Last access March 3, 2022 CABI 1935-2019 - 5659)
A. obliqua,-48.00,-10.50	CABI	https://www.cabidigitallibrary.org (Last access March 3, 2022 CABI 1935-2019 - 5659)
A. obliqua,-73.25,4.00	CABI	https://www.cabidigitallibrary.org (Last access March 3, 2022 CABI 1935-2019 - 5659)
A. obliqua,-76.57,3.37	GBIF	https://doi.org/10.15468/dl.9 3h5p7
A. obliqua,-76.95,4.25	GBIF	https://doi.org/10.15468/dl.9 3h5p7
A. obliqua,-76.52,2.93	GBIF	https://doi.org/10.15468/dl.9 3h5p7
A. obliqua,-75.61,4.99	GBIF	https://doi.org/10.15468/dl.9 3h5p7
A. obliqua,-76.09,4.55	GBIF	https://doi.org/10.15468/dl.9 3h5p7
A. obliqua,-76.78,3.58	GBIF	https://doi.org/10.15468/dl.9 3h5p7
A. obliqua,-76.33,3.51	GBIF	https://doi.org/10.15468/dl.9 3h5p7
A. obliqua,-75.95,4.25	GBIF	https://doi.org/10.15468/dl.9 3h5p7
A. obliqua,-74.31,3.68	GBIF	https://doi.org/10.15468/dl.9 3h5p7
A. obliqua,-75.58,6.19	GBIF	https://doi.org/10.15468/dl.9 3h5p7

A. obliqua,-58.00,-23.33	CABI	https://www.cabidigitallibrary.org (Last access March 3, 2022 CABI 1935-2019 - 5659)
A. obliqua,-75.25,-10.00	CABI	https://www.cabidigitallibrary.org (Last access March 3, 2022 CABI 1935-2019 - 5659)
A. obliqua,-70.09,-12.56	GBIF	https://doi.org/10.15468/dl.93h5p7
A. obliqua,-80.00,9.00	CABI	https://www.cabidigitallibrary.org (Last access March 3, 2022 CABI 1935-2019 - 5659)
A. obliqua,-79.39,9.14	GBIF	https://doi.org/10.15468/dl.93h5p7
A. obliqua,-63.65,-17.50	GBIF	https://doi.org/10.15468/dl.93h5p7
A. obliqua,-64.54,-17.20	GBIF	https://doi.org/10.15468/dl.93h5p7
A. obliqua,-102.00,23.00	CABI	https://www.cabidigitallibrary.org (Last access March 3, 2022 CABI 1935-2019 - 5659)
A. obliqua,-91.51,18.21	GBIF	https://doi.org/10.15468/dl.93h5p7
A. obliqua,-90.64,18.58	GBIF	https://doi.org/10.15468/dl.93h5p7
A. obliqua,-95.17,18.50	GBIF	https://doi.org/10.15468/dl.93h5p7
A. obliqua,-98.96,21.72	GBIF	https://doi.org/10.15468/dl.93h5p7
A. obliqua,-94.19,16.31	GBIF	https://doi.org/10.15468/dl.93h5p7
A. obliqua,-97.84,22.23	GBIF	https://doi.org/10.15468/dl.93h5p7
A. obliqua,-88.75,17.25	CABI	https://www.cabidigitallibrary.org (Last access March 3, 2022 CABI 1935-2019 - 5659)
A. obliqua,-88.92,13.83	CABI	https://www.cabidigitallibrary.org (Last access March 3, 2022 CABI 1935-2019 - 5659)
A. obliqua,-90.25,15.50	CABI	https://www.cabidigitallibrary.org (Last access March 3, 2022 CABI 1935-2019 - 5659)
A. obliqua,-86.50,15.00	CABI	https://www.cabidigitallibrary.org (Last access March 3, 2022 CABI 1935-2019 - 5659)
A. obliqua,-85.00,13.00	CABI	https://www.cabidigitallibrary.org (Last access March 3, 2022 CABI 1935-2019 - 5659)
A. obliqua,-78.25,-1.25	CABI	https://www.cabidigitallibrary.org (Last access March 3, 2022 CABI 1935-2019 - 5659)

A. obliqua,-53.00,4.00	CABI	https://www.cabidigitallibrary.org (Last access March 3, 2022 CABI 1935-2019 - 5659)	
A. obliqua,-59.00,5.00	CABI	https://www.cabidigitallibrary.org (Last access March 3, 2022 CABI 1935-2019 - 5659)	
A. obliqua,-56.00,4.00	CABI	https://www.cabidigitallibrary.org (Last access March 3, 2022 CABI 1935-2019 - 5659)	
A. obliqua,-66.00,8.00	CABI	https://www.cabidigitallibrary.org (Last access March 3, 2022 CABI 1935-2019 - 5659)	
A. obliqua,-77.80,8.35	SV	https://mida.gob.pa/sanidad-vegetal (Last access March 10, 2022)	
A. obliqua,-78.06,8.59	CABI	https://www.cabidigitallibrary.org (Last access March 3, 2022 CABI 1935-2019 - 5659)	
A. obliqua,-78.19,8.83	SV	https://mida.gob.pa/sanidad-vegetal (Last access March 10, 2022)	
A. obliqua,-79.71,9.26	GBIF	https://doi.org/10.15468/dl.9 3h5p7	
A. obliqua,-80.23,8.38	SV	https://mida.gob.pa/sanidad-vegetal (Last access March 10, 2022)	
A. obliqua,-80.23,8.72	SV	https://mida.gob.pa/sanidad-vegetal (Last access March 10, 2022)	
A. obliqua,-80.42,7.67	SV	https://mida.gob.pa/sanidad-vegetal (Last access March 10, 2022)	
A. obliqua,-80.49,8.63	SV	https://mida.gob.pa/sanidad-vegetal (Last access March 10, 2022)	
A. obliqua,-80.62,8.12	SV	https://mida.gob.pa/sanidad-vegetal (Last access March 10, 2022)	
A. obliqua,-80.75,7.85	SV	https://mida.gob.pa/sanidad-vegetal (Last access March 10, 2022)	
A. obliqua,-80.75,8.44	SV	https://mida.gob.pa/sanidad-vegetal (Last access March 10, 2022)	
A. obliqua,-82.17,8.87	SV	https://mida.gob.pa/sanidad-vegetal (Last access March 10, 2022)	
A. obliqua,-82.51,8.65	SV	https://mida.gob.pa/sanidad-vegetal (Last access March 10, 2022)	
A. obliqua,-82.52,9.45	SV	https://mida.gob.pa/sanidad-vegetal (Last access March 10, 2022)	
A. obliqua,-82.87,9.59	GBIF	https://doi.org/10.15468/dl.9 3h5p7	
A. obliqua,-50.58,-29.45	SPECIES	http://www.splink.org.br/	speciesLink network, 16-Jun-2022 12:51, specieslink.net/search
A. obliqua,-58.01,-28.59	SPECIES	http://www.splink.org.br/	speciesLink network, 16-Jun-2022 12:51, specieslink.net/search
A. obliqua,-65.43,-24.04	SPECIES	http://www.splink.org.br/	speciesLink network, 16-Jun-2022 12:51, specieslink.net/search
A.striata,-75.08032200000,6.90975700000	Colección:	2007_Giraldo_Monitoramento de moscas-das-frutas (Diptera, Tephritidae, Anastrepha).	
A.striata,-75.45490300000,6.37931600000	Colección:	2007_Giraldo_Monitoramento de moscas-das-frutas (Diptera, Tephritidae, Anastrepha)	

A.striata,-75.03629300000,6.30765200000	Colección:	2007_Giraldo_Monitoramento de moscas-das-frutas (Diptera, Tephritidae, Anastrepha).
A.striata,-75.82992600000,6.56398000000	Colección:	2007_Giraldo_Monitoramento de moscas-das-frutas (Diptera, Tephritidae, Anastrepha).
A.striata,-75.46661400000,6.65246000000	Colección:	2007_Giraldo_Monitoramento de moscas-das-frutas (Diptera, Tephritidae, Anastrepha).
A.striata,-76.88667300000,6.59695800000	Colección:	2007_Giraldo_Monitoramento de moscas-das-frutas (Diptera, Tephritidae, Anastrepha).
A.striata,-75.01644100000,6.59905200000	Colección:	2007_Giraldo_Monitoramento de moscas-das-frutas (Diptera, Tephritidae, Anastrepha).
A.striata,-75.54995300000,2.38863200000	Colección:	2007_Giraldo_Monitoramento de moscas-das-frutas (Diptera, Tephritidae, Anastrepha).
A.striata,-74.20185400000,11.23678600000	Colección:	2007_Giraldo_Monitoramento de moscas-das-frutas (Diptera, Tephritidae, Anastrepha).
A.striata,-73.62168900000,4.13315000000	Colección:	2007_Giraldo_Monitoramento de moscas-das-frutas (Diptera, Tephritidae, Anastrepha).
A.striata,-72.50716200000,7.91092700000	Colección:	2007_Giraldo_Monitoramento de moscas-das-frutas (Diptera, Tephritidae, Anastrepha).
A.striata,-72.47909000000,7.57827200000	Colección:	2007_Giraldo_Monitoramento de moscas-das-frutas (Diptera, Tephritidae, Anastrepha).
A.striata,-74.88671600000,4.15247500000	Colección:	2007_Giraldo_Monitoramento de moscas-das-frutas (Diptera, Tephritidae, Anastrepha).
A.striata,-74.88974100000,5.20459700000	Colección:	2012_ sarmiento C.E. et al_ Anastrepha (DIPTERA: TEPHRITIDAE) Y SUS ASOCIADOS:
A.striata,-73.36799200000,5.07547500000	Colección:	2012_ sarmiento C.E. et al_ Anastrepha (DIPTERA: TEPHRITIDAE) Y SUS ASOCIADOS:
A.striata,-72.68598600000,6.33493400000	Colección:	2012_ sarmiento C.E. et al_ Anastrepha (DIPTERA: TEPHRITIDAE) Y SUS ASOCIADOS:
A.striata,-74.09611100000,4.64805600000	Colección:	2012_ sarmiento C.E. et al_ Anastrepha (DIPTERA: TEPHRITIDAE) Y SUS ASOCIADOS:
A.striata,-73.94940400000,4.40363900000	Colección:	2012_ sarmiento C.E. et al_ Anastrepha (DIPTERA: TEPHRITIDAE) Y SUS ASOCIADOS:
A.striata,-74.39249200000,4.65674000000	GBIF	https://doi.org/10.15468/dl.frjaxe
A.striata,-76.29824600000,3.53975400000	GBIF	https://doi.org/10.15468/dl.frjaxe
A.striata,-75.93386900000,4.26920500000	Colección:	2012_ sarmiento C.E. et al_ Anastrepha (DIPTERA: TEPHRITIDAE) Y SUS ASOCIADOS:
A.striata,-76.19892100000,4.08682000000	GBIF	https://doi.org/10.15468/dl.frjaxe

A.striata,-72.94090700000,5.77458700000	Colección:	Castañeda, María del Rosario, Osorio F, Agronomía Colombiana, 28(2), 264-272.
A.striata,-75.36386200000,5.97506900000	Colección:	Castañeda, María del Rosario, Osorio F, Agronomía Colombiana, 28(2), 264-272.
A.striata,-74.63879200000,4.46231900000	Colección:	Castañeda, María del Rosario, Osorio F, Agronomía Colombiana, 28(2), 264-272.
A.striata,-74.38344800000,4.33061900000	Colección:	Castañeda, María del Rosario, Osorio F, Agronomía Colombiana, 28(2), 264-272.
A.striata,-74.16138500000,5.13135300000	Colección:	Castañeda, María del Rosario, Osorio F, Agronomía Colombiana, 28(2), 264-272.
A.striata,-73.78566700000,5.87790300000	Colección:	Castañeda, María del Rosario, Osorio F, Agronomía Colombiana, 28(2), 264-272.
A.striata,-74.98333300000,3.58333300000	Colección:	Castañeda, María del Rosario, Osorio F, Agronomía Colombiana, 28(2), 264-272.
A.striata,-76.51425000000,3.80703000000	GBIF	https://doi.org/10.15468/dl.frjaxe
A.striata,-76.49350000000,7.69968000000	Colección:	Castañeda, María del Rosario, Osorio F, Agronomía Colombiana, 28(2), 264-272.
A.striata,-76.05015000000,4.66771000000	Colección:	Castañeda, María del Rosario, Osorio F, Agronomía Colombiana, 28(2), 264-272.
A.striata,-75.19028000000,4.09556000000	Colección:	Castañeda, María del Rosario, Osorio F, Agronomía Colombiana, 28(2), 264-272.
A.striata,-76.58880000000,4.41789000000	Colección:	Castañeda, María del Rosario, Osorio F, Agronomía Colombiana, 28(2), 264-272.
A.striata,-74.47483300000,5.05977800000	Colección:	Castañeda, María del Rosario, Osorio F, Agronomía Colombiana, 28(2), 264-272.
A.striata,-75.06660000000,4.91660000000	Colección:	Castañeda, María del Rosario, Osorio F, Agronomía Colombiana, 28(2), 264-272.
A.striata,-75.83216700000,8.71145300000	Colección:	ICA-Boletines epidemiológicos Plan Nacional Moscas de la Fruta; iv trimestre 2015
A.striata,-75.84060100000,9.01638200000	Colección:	ICA-Boletines epidemiológicos Plan Nacional Moscas de la Fruta; iv trimestre 2015
A.striata,-75.42444600000,9.66544000000	Colección:	ICA-Boletines epidemiológicos Plan Nacional Moscas de la Fruta; iv trimestre 2015
A.striata,-75.46716100000,8.20190600000	Colección:	ICA-Boletines epidemiológicos Plan Nacional Moscas de la Fruta; iv trimestre 2015
A.striata,-72.25103600000,11.39039500000	Colección:	ICA-Boletines epidemiológicos Plan Nacional Moscas de la Fruta; iv trimestre 2015
A.striata,-72.85024700000,10.88772800000	Colección:	ICA-Boletines epidemiológicos Plan Nacional Moscas de la Fruta; iv trimestre 2015

A.striata,-72.99118000000,10.59342200000	Colección:	ICA-Boletines epidemiológicos Plan Nacional Moscas de la Fruta; iv trimestre 2015
A.striata,-72.66581700000,7.37463400000	Colección:	ICA-Boletines epidemiológicos Plan Nacional Moscas de la Fruta; iv trimestre 2015
A.striata,-74.92326100000,10.25819500000	Colección:	ICA-Boletines epidemiológicos Plan Nacional Moscas de la Fruta; iv trimestre 2015
A.striata,-75.30921400000,10.58380500000	Colección:	ICA-Boletines epidemiológicos Plan Nacional Moscas de la Fruta; iv trimestre 2015
A.striata,-73.37168800000,5.70711900000	Colección:	ICA-Boletines epidemiológicos Plan Nacional Moscas de la Fruta; iv trimestre 2015
A.striata,-76.52006700000,2.95710000000	Colección:	ICA-Boletines epidemiológicos Plan Nacional Moscas de la Fruta; iv trimestre 2015
A.striata,-76.95774000000,2.66246100000	Colección:	ICA-Boletines epidemiológicos Plan Nacional Moscas de la Fruta; iv trimestre 2015
A.striata,-73.95679800000,3.41815000000	Colección:	ICA-Boletines epidemiológicos Plan Nacional Moscas de la Fruta; iv trimestre 2015
A.striata,-77.67642500000,0.83267900000	Colección:	ICA-Boletines epidemiológicos Plan Nacional Moscas de la Fruta; iv trimestre 2015
A.striata,-78.75566700000,1.67524600000	Colección:	ICA-Boletines epidemiológicos Plan Nacional Moscas de la Fruta; iv trimestre 2015
A.striata,-77.12716800000,1.58076000000	Colección:	ICA-Boletines epidemiológicos Plan Nacional Moscas de la Fruta; iv trimestre 2015
A.striata,-77.32179900000,1.15113000000	Colección:	ICA-Boletines epidemiológicos Plan Nacional Moscas de la Fruta; iv trimestre 2015
A.striata,-69.93980800000,-4.21910200000	Colección:	ICA-Boletines epidemiológicos Plan Nacional Moscas de la Fruta; iv trimestre 2015
A.striata,-70.46346800000,6.93217900000	Colección:	ICA-Boletines epidemiológicos Plan Nacional Moscas de la Fruta; iv trimestre 2015
A.striata,-71.17143900000,6.89702000000	Colección:	ICA-Boletines epidemiológicos Plan Nacional Moscas de la Fruta; iv trimestre 2015
A.striata,-71.85863000000,6.99601900000	Colección:	ICA-Boletines epidemiológicos Plan Nacional Moscas de la Fruta; iv trimestre 2015
A.striata,-67.65223100000,5.69736600000	Colección:	ICA-Boletines epidemiológicos Plan Nacional Moscas de la Fruta; iv trimestre 2015
A.striata,-67.50047200000,6.18940700000	Colección:	ICA-Boletines epidemiológicos Plan Nacional Moscas de la Fruta; iv trimestre 2015
A.striata,-75.61863100000,4.52587400000	GBIF	https://doi.org/10.15468/dl.frjaxe
A.striata,-75.66645700000,4.81886500000	GBIF	https://doi.org/10.15468/dl.frjaxe

A.striata,-75.96585400000,5.08053300000	Colección:	ICA-Boletines epidemiológicos Plan Nacional Moscas de la Fruta; iv trimestre 2015
A.striata,-76.88412000000,0.41398000000	Colección:	ICA-Boletines epidemiológicos Plan Nacional Moscas de la Fruta; iv trimestre 2015
A.striata,-75.33304500000,1.56190200000	Colección:	ICA-Boletines epidemiológicos Plan Nacional Moscas de la Fruta; iv trimestre 2015
A.striata,-75.72849100000,1.49458400000	Colección:	ICA-Boletines epidemiológicos Plan Nacional Moscas de la Fruta; iv trimestre 2015
A.striata,-75.96255100000,1.33179500000	Colección:	ICA-Boletines epidemiológicos Plan Nacional Moscas de la Fruta; iv trimestre 2015
A.striata,-88.75000000000,17.25000000000	CABI	https://www.cabidigitallibrary.org (Last access March 3, 2022 CABI 1943-2020 - 5657)
A.striata,-84.00000000000,10.00000000000	GBIF	https://doi.org/10.15468/dl.frjaxe
A.striata,-82.95627500000,9.51934900000	SV	https://mida.gob.pa/sanidad-vegetal
A.striata,-83.74200500000,10.64405000000	GBIF	https://doi.org/10.15468/dl.frjaxe
A.striata,-84.48510100000,10.20136100000	GBIF	https://doi.org/10.15468/dl.frjaxe
A.striata,-83.06352300000,9.82279500000	GBIF	https://doi.org/10.15468/dl.frjaxe
A.striata,-82.77110500000,8.89594200000	GBIF	https://doi.org/10.15468/dl.frjaxe
A.striata,-85.05995200000,9.86709500000	GBIF	https://doi.org/10.15468/dl.frjaxe
A.striata,-85.63359700000,10.13425900000	GBIF	https://doi.org/10.15468/dl.frjaxe
A.striata,-83.22732800000,8.68065600000	GBIF	https://doi.org/10.15468/dl.frjaxe
A.striata,-85.24638400000,10.33497400000	GBIF	https://doi.org/10.15468/dl.frjaxe
A.striata,-84.00818700000,10.43195700000	GBIF	https://doi.org/10.15468/dl.frjaxe
A.striata,-83.52255000000,8.57016300000	GBIF	https://doi.org/10.15468/dl.frjaxe
A.striata,-83.59565200000,9.39570100000	GBIF	https://doi.org/10.15468/dl.frjaxe
A.striata,-84.79662000000,10.27672300000	GBIF	https://doi.org/10.15468/dl.frjaxe

A.striata,-83.01452800000,9.03118400000	GBIF	https://doi.org/10.15468/dl.frjaxe
A.striata,-83.69493600000,9.97397900000	GBIF	https://doi.org/10.15468/dl.frjaxe
A.striata,-85.35191300000,10.77678400000	GBIF	https://doi.org/10.15468/dl.frjaxe
A.striata,-85.02321800000,10.57558900000	GBIF	https://doi.org/10.15468/dl.frjaxe
A.striata,-85.57695300000,11.03365700000	GBIF	https://doi.org/10.15468/dl.frjaxe
A.striata,-90.25000000000,15.50000000000	CABI	https://www.cabidigitallibrary.org (Last access March 3, 2022 CABI 1943-2020 - 5657)
A.striata,-86.50000000000,15.00000000000	CABI	https://www.cabidigitallibrary.org (Last access March 3, 2022 CABI 1943-2020 - 5657)
A.striata,-87.00363100000,14.01429900000	GBIF	https://doi.org/10.15468/dl.frjaxe
A.striata,-102.00000000000,23.00000000000	CABI	https://www.cabidigitallibrary.org (Last access March 3, 2022 CABI 1943-2020 - 5657)
A.striata,-98.05000300000,25.95999900000	GBIF	https://doi.org/10.15468/dl.frjaxe
A.striata,-99.09305300000,18.98480200000	GBIF	https://doi.org/10.15468/dl.frjaxe
A.striata,-103.39070300000,20.66824300000	GBIF	https://doi.org/10.15468/dl.frjaxe
A.striata,-92.39900000000,15.01100000000	GBIF	https://doi.org/10.15468/dl.frjaxe
A.striata,-92.18300000000,14.81700000000	GBIF	https://doi.org/10.15468/dl.frjaxe
A.striata,-85.00000000000,13.00000000000	CABI	https://www.cabidigitallibrary.org (Last access March 3, 2022 CABI 1943-2020 - 5657)
A.striata,-80.00000000000,9.00000000000	CABI	https://www.cabidigitallibrary.org (Last access March 3, 2022 CABI 1943-2020 - 5657)
A.striata,-63.65000200000,-18.12999900000	GBIF	https://doi.org/10.15468/dl.frjaxe
A.striata,-64.53731000000,-17.19727700000	GBIF	https://doi.org/10.15468/dl.frjaxe
A.striata,-64.86225000000,-17.04622000000	GBIF	https://doi.org/10.15468/dl.frjaxe
A.striata,-63.65335000000,-17.49542000000	GBIF	https://doi.org/10.15468/dl.frjaxe

A.striata,-63.37363000000,-17.99858000000	GBIF	https://doi.org/10.15468/dl.frjaxe
A.striata,-55.00000000000,-10.00000000000	CABI	https://www.cabidigitallibrary.org (Last access March 3, 2022 CABI 1943-2020 - 5657)
A.striata,-70.00000000000,-9.00000000000	CABI	https://www.cabidigitallibrary.org (Last access March 3, 2022 CABI 1943-2020 - 5657)
A.striata,-52.00000000000,1.00000000000	CABI	https://www.cabidigitallibrary.org (Last access March 3, 2022 CABI 1943-2020 - 5657)
A.striata,-64.50000000000,-3.75000000000	CABI	https://www.cabidigitallibrary.org (Last access March 3, 2022 CABI 1943-2020 - 5657)
A.striata,-49.63623000000,-15.58071000000	CABI	https://www.cabidigitallibrary.org (Last access March 3, 2022 CABI 1943-2020 - 5657)
A.striata,-45.00000000000,-5.00000000000	CABI	https://www.cabidigitallibrary.org (Last access March 3, 2022 CABI 1943-2020 - 5657)
A.striata,-55.00000000000,-20.50000000000	CABI	https://www.cabidigitallibrary.org (Last access March 3, 2022 CABI 1943-2020 - 5657)
A.striata,-44.00000000000,-18.00000000000	CABI	https://www.cabidigitallibrary.org (Last access March 3, 2022 CABI 1943-2020 - 5657)
A.striata,-53.00000000000,-4.00000000000	CABI	https://www.cabidigitallibrary.org (Last access March 3, 2022 CABI 1943-2020 - 5657)
A.striata,-42.33333000000,-7.25000000000	CABI	https://www.cabidigitallibrary.org (Last access March 3, 2022 CABI 1943-2020 - 5657)
A.striata,-63.00000000000,-11.00000000000	CABI	https://www.cabidigitallibrary.org (Last access March 3, 2022 CABI 1943-2020 - 5657)
A.striata,-61.25000000000,2.25000000000	CABI	https://www.cabidigitallibrary.org (Last access March 3, 2022 CABI 1943-2020 - 5657)
A.striata,-49.00000000000,-22.00000000000	CABI	https://www.cabidigitallibrary.org (Last access March 3, 2022 CABI 1943-2020 - 5657)
A.striata,-48.00000000000,-10.50000000000	CABI	https://www.cabidigitallibrary.org (Last access March 3, 2022 CABI 1943-2020 - 5657)
A.striata,-73.25000000000,4.00000000000	CABI	https://www.cabidigitallibrary.org (Last access March 3, 2022 CABI 1943-2020 - 5657)
A.striata,-75.33640000000,4.40909000000	GBIF	https://doi.org/10.15468/dl.frjaxe
A.striata,-77.00000000000,3.81000000000	GBIF	https://doi.org/10.15468/dl.frjaxe
A.striata,-76.91000400000,4.28000000000	GBIF	https://doi.org/10.15468/dl.frjaxe
A.striata,-76.57000000000,3.37000000000	GBIF	https://doi.org/10.15468/dl.frjaxe

A.striata,-76.77999900000,3.58000000000	GBIF	https://doi.org/10.15468/dl.frjaxe
A.striata,-75.62549200000,6.15710200000	GBIF	https://doi.org/10.15468/dl.frjaxe
A.striata,-78.25000000000,-1.25000000000	CABI	https://www.cabidigitallibrary.org (Last access March 3, 2022 CABI 1943-2020 - 5657)
A.striata,-53.00000000000,4.00000000000	CABI	https://www.cabidigitallibrary.org (Last access March 3, 2022 CABI 1943-2020 - 5657)
A.striata,-52.33110000000,4.84720000000	GBIF	https://doi.org/10.15468/dl.frjaxe
A.striata,-58.93020000000,4.86040000000	GBIF	https://doi.org/10.15468/dl.frjaxe
A.striata,-58.00000000000,-23.33333000000	CABI	https://www.cabidigitallibrary.org (Last access March 3, 2022 CABI 1943-2020 - 5657)
A.striata,-75.25000000000,-10.00000000000	CABI	https://www.cabidigitallibrary.org (Last access March 3, 2022 CABI 1943-2020 - 5657)
A.striata,-71.40000200000,-12.89000000000	GBIF	https://doi.org/10.15468/dl.frjaxe
A.striata,-74.93403000000,-9.61786500000	GBIF	https://doi.org/10.15468/dl.frjaxe
A.striata,-56.00000000000,4.00000000000	CABI	https://www.cabidigitallibrary.org (Last access March 3, 2022 CABI 1943-2020 - 5657)
A.striata,-66.00000000000,8.00000000000	CABI	https://www.cabidigitallibrary.org (Last access March 3, 2022 CABI 1943-2020 - 5657)
A.striata,-66.58970000000,6.42380000000	CABI	https://www.cabidigitallibrary.org (Last access March 3, 2022 CABI 1943-2020 - 5657)
A.striata,-71.63330000000,10.63330000000	GBIF	https://doi.org/10.15468/dl.frjaxe
A.striata,-89.32000000000,13.93000000000	GBIF	https://doi.org/10.15468/dl.frjaxe
A.striata,-77.81247500000,8.29205000000	SV	https://mida.gob.pa/sanidad-vegetal
A.striata,-78.01024400000,8.54738300000	SV	https://mida.gob.pa/sanidad-vegetal
A.striata,-78.19135800000,8.83184600000	SV	https://mida.gob.pa/sanidad-vegetal
A.striata,-79.68582300000,9.29644500000	SV	https://mida.gob.pa/sanidad-vegetal
A.striata,-80.28973600000,8.71511800000	SV	https://mida.gob.pa/sanidad-vegetal

A.striata,-80.55315700000,8.49522700000	SV	https://mida.gob.pa/sanidad-vegetal	
A.striata,-80.57967300000,8.80280600000	SV	https://mida.gob.pa/sanidad-vegetal	
A.striata,-80.86193800000,8.00792900000	SV	https://mida.gob.pa/sanidad-vegetal	
A.striata,-82.24505100000,8.99694500000	SV	https://mida.gob.pa/sanidad-vegetal	
A.striata,-82.51855300000,8.68478900000	SV	https://mida.gob.pa/sanidad-vegetal	
A.striata,-82.57429800000,9.35321400000	SV	https://mida.gob.pa/sanidad-vegetal	
A.striata,-51.46110153000,-29.68860000000	SPECIES	http://www.splink.org.br/	speciesLink network, 16-Jun-2022 12:13, specieslink.net/search
A.striata,-80.60981476000,-5.19526700000	SV	https://mida.gob.pa/sanidad-vegetal	

Table S2: Main environmental variables that contribute to the model for (*A. grandis, A. serpentina, A. obliqua, A. striata*)

1. **Main environmental variables that contribute to the MaxEnt model for *A. grandis*.**

Set 1	Set 2	Set 3	Set 4	Set 5	Set 6	Set 7	Set 8
All	EVI_range	EVI_min	EVI_range	All variables related to NDVI	All variables related to EVI	All variables related to LST	All variables related to precipitation
	LST_range	NDVI_max	LST_min				
	NDVI_range	NDVI_range	PRECIP_range				
	PRECIP_range	PRECIP_range	NDVI_max				
	PRECIP_max	EVI_range	NDVI_range				
	PRECIP_mean		PRECIP_min				
	PRECIP_min		LST_range				

2. Main environmental variables that contribute to the MaxEnt model for *A. serpentina*.

Set 1	Set 2	Set 3	Set 4	Set 5	Set 6	Set 7	Set 8
All	EVI_range	EVI_range	EVI_max	All variables related to NDVI	All variables related to EVI	All variables related to LST	All variables related to precipitation
	LST_max	NDVI_min	EVI_range				
	LST_mean	PRECIP_range	LST_min				
	LST_min	EVI_mean	LST_range				
	LST_range	LST_range	NDVI_range				
	NDVI_range	LST_min	PRECIP_min				
	PRECIP_max	EVI_max	PRECIP_range				
	PRECIP_mean						
	PRECIP_min						
	PRECIP_range						

3. Main environmental variables that contribute to the MaxEnt model for *A. obliqua*.

Set 1	Set 2	Set 3	Set 4	Set 5	Set 6	Set 7	Set 8
All	EVI_range	EVI_range	EVI_max	All variables related to NDVI	All variables related to EVI	All variables related to LST	All variables related to precipitation
	LST_max	PRECIP_range	EVI_range				
	LST_mean	PRECIP_min	LST_min				
	LST_min	LST_min	LST_range				
	LST_range	NDVI_range	NDVI_range				
	NDVI_range		PRECIP_min				
	PRECIP_max		PRECIP_range				
	PRECIP_mean						
	PRECIP_min						

4. Main environmental variables that contribute to the MaxEnt model for *A. striata*.

Set 1	Set 2	Set 3	Set 4	Set 5	Set 6	Set 7	Set 8
All	EVI_range	LST_range	EVI_max	All variables related to NDVI	All variables related to EVI	All variables related to LST	All variables related to precipitation
	LST_max	LST_min	EVI_range				
	LST_mean	EVI_max	LST_min				
	LST_min	PRECIP_min	LST_range				
	LST_range		NDVI_range				
	NDVI_range		PRECIP_min				
	PRECIP_max		PRECIP_range				
	PRECIP_mean						
	PRECIP_min						
	PRECIP_range						

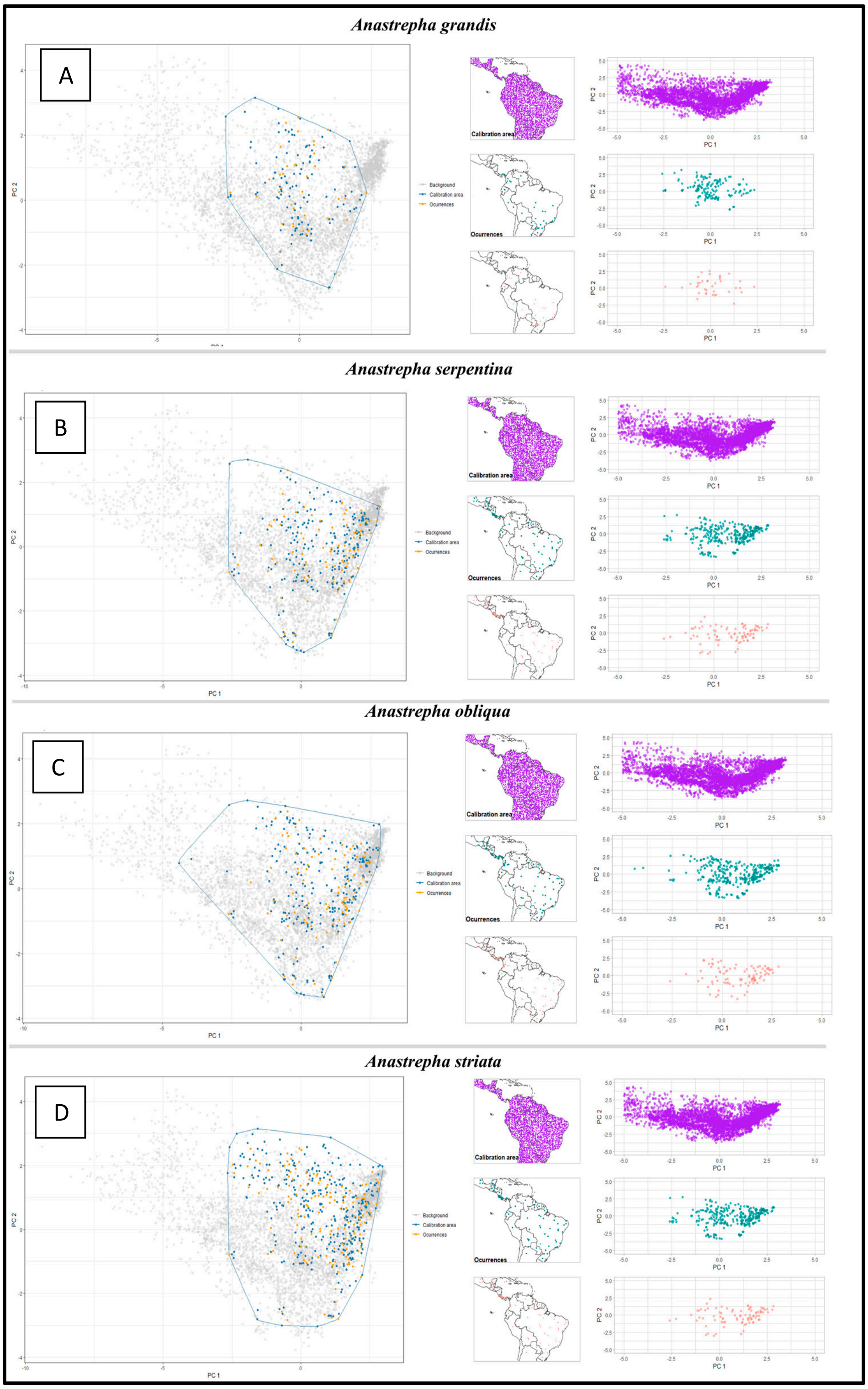

Figure S1. Analysis of the environmental space represented by presence records, calibration area and projection area.
